# Olive Oil Injections in Constipation

**Published:** 1904-10-22

**Authors:** 


### OLIVE-OIL INJECTIONS IN CONSTIPATION.

Dr. George Herschell[1] advises the use of olive-oil injections in certain types of constipation. He also finds this method valuable in muco-membranous colitis, as it considerably reduces the secretion of mucus. The selection of suitable cases is of the first importance, as neither olive-oil injections nor any one form of treatment can be wisely employed as a routine measure. The cases of constipation in which the injections may be tried are (1) cases depending on chronic colitis; (2) constipation associated with spasm of the bowel, as is often found in neurasthenia; and (3) cases of atony of the bowel in order to secure a daily evacuation until the effects of electrical treatment have had time to manifest themselves. Hardly less important than a wise selection of cases is the manner of using the injections. The oil should be warmed, and in quantities ranging from three to ten ounces should be introduced into the rectum at bed time and retained until the following morning. It should not be driven forcibly into the bowel from a syringe, for this is apt to excite immediate peristalsis and consequent expulsion of the oil. Rather it should be allowed to pass into the rectum slowly and under a low pressure, a result which can be best obtained by placing the oil in a funnel-shaped vessel connected by indiarubber tubing with a rectal tube. On raising the funnel the oil enters the rectum under the influence of gravity. The apparatus made for the author by Allen and Hanbury is well adapted for self-use.

[1] Lancet, Oct. 1, 1904.

